# Low-dose sclerotherapy with lauromacrogol in the treatment of infantile hemangiomas: A retrospective analysis of 368 cases

**DOI:** 10.3389/fonc.2022.1014465

**Published:** 2022-11-08

**Authors:** Changfeng Wang, Jiali Sun, Lei Guo, Dan Song, Xin Zhang, Zhuang Liu, Liang Wang

**Affiliations:** Department of Vascular Anomalies and Interventional Radiology, Children’s Hospital Affiliated to Shandong University, Jinan, Shandong, China

**Keywords:** infantile hemangioma, treatment, lauromacrogol sclerotherapy, intralesional injection, vascular endothelial growth factor

## Abstract

**Background:**

Intralesional injection of lauromacrogol has proven to be an efficient treatment method for infantile hemangioma. This study aims to explore a novel injection therapy and evaluate its efficacy and complications.

**Methods:**

The medical records of 368 children with infantile hemangioma who received a lauromacrogol injection from January 2021 to April 2022 were retrospectively analyzed. All patients were reviewed every 4 weeks, and their condition was assessed according to symptoms and medical records. The patient’s age, lesion type, location, size and thickness, lesion photographs, ultrasound, and complications were recorded.

**Results:**

Among the 368 infants who accept sclerotherapy with lauromacrogol, 226(61.4%)achieved excellent regression. In total, 108(29.4%)cases achieved good regression. 24(6.5%)achieved complete moderate regression. 10(2.7%)achieved poor regression. The reported incidence of adverse events was 4.9% and severe complications were not observed. Before and after three courses of treatment, the median vascular endothelial growth factor levels were 104.12 pg/ml and 28.982 pg/ml. There was a significant difference between the two groups (P=0.0043).

**Conclusions:**

The results showed that this novel injection therapy a safe and effective treatment method. The therapy accelerated the regression of infantile hemangiomas without serious complications.

## Introduction

Infantile hemangiomas (IHs) are one of the commonest infantile benign angiogenic tumors, with an incidence of 2–10% (1). The lesion is usually diagnosed within the first few weeks of life and IHs have a distinct natural history involving three stages. Almost all IHs undergo a proliferative, stable and slow spontaneous degeneration stage. IHs grow rapidly during the first 3 to 5 months of life and may continue until 9 to 12 months of age, followed by a slow period of involution (2).

Degeneration begins at about 1 year of age and can take several years to fully regress.About 95% of IHs will reach complete regression by 10 to 12 years. Although IHs are characterized by regular growth and regression and most do not require treatment, complications may lead to functional or cosmetic disability (2). In order to avoid any complications, the treatment of the hemangioma may be necessary in selected cases.

The various methods for treating IHs include the use of propranolol, atenolol, corticosteroids, sclerotherapy, laser therapy, and embolization (3–5). Propranolol, a nonselective beta-blocker, has become the first-line drug for treating IHs, especially for high-risk hemangiomas, including giant hemangiomas with a risk of ulceration, bleeding, and infection (4, 6). However, long-term continued oral propranolol may have side effects, including low blood pressure, bronchospasm, and sleep disturbance. Adverse reactions can cause parents psychological stress, thereby reducing compliance with treatment (7). Sclerotherapy may be an option for some patients with limited tumors and difficulty feeding. Sclerotherapy has been widely used in the treatment of IHs and vascular malformations, and lauromacrogol (polyoxyethylene laurel alcohol ether, lauromacrogol) is a is a sclerosing agent approved in China since 2018 (8–10). Similar to polidocanol, it promotes vascular fibrosis and thrombosis and has the effect of low toxicity, hypoallergic and mild anesthesia. It is effective when used for the sclerotherapy of IHs and vascular malformations, but there are still some complications including: swelling, ulceration, infection and pain. The purpose of our study was to explore a novel sclerosant injection method and to evaluate retrospectively the safety of intralesional lauromacrogol injection for therapeutic intervention in children with IHs.

## Methods

We conducted a retrospective study of 509 patients with IHs managed by the Department of Vascular Anomalies and Interventional Radiology, Children’s Hospital Affiliated to Shandong University. They all received an intralesional injection of lauromacrogol, 368 patients who met the final criteria were retrospectively analyzed in the study. This study complies with the Declaration of Helsinki and was approved by the Ethics Committee of the Children’s Hospital of Shandong University. All patients in this study signed informed consent from their guardians.

The inclusion criteria were (1) IHs treated with lauromacrogol from January 1, 2021 until April 31, 2022. (2) All cases were diagnosed by clinical symptoms, physical examination and color Doppler. (3) Not received new treatments during sclerotherapy. (4) IHs are not accompanied by major complications such as ulceration, bleeding, dysfunction, and disfigurement. We recorded photographs and ultrasound images at the patient’s initial visit and at each outpatient review. The following information was extracted from each patient’s medical record: age, sex, anatomical location, depth, and size of the lesion, vascular endothelial growth factor (VEGF) level, and complications.

**Figure 1:**
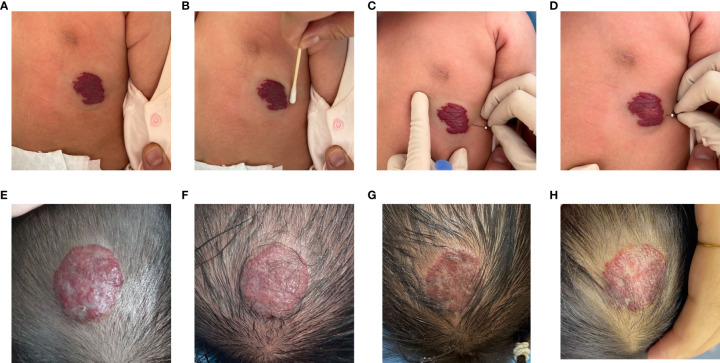
The procedure of intralesional injection **(A–D)** and a 4-month-old girl with a head hemangioma received intralesional injection **(E–H)**.**(A)** Baseline prior to initiation of intralesional injection. **(B)** Disinfection of the surface and perimeter of the lesion. **(C)** Needle is inserted through the normal tissue surrounding the lesion and punctured the middle layer of the lesion. **(D)** Suction before pushing the drug and then injecting the drug slowly until the surface of the tumor was slightly white,if there was no blood backflow.**(E)** Mixed infantile hemangioma of the head before the first injection. **(F)** First course of injections. **(G)** Second course of injections. **(H)** Third course of injections.

We took the following measures:(1) Patients were treated with surface anesthesia and 5% compound lidocaine cream on the surface of the lesion and within 1cm of the surrounding area 1 hour prior to injection treatment.(2) 1% lauromacrogol was diluted 1:10 with normal saline. (3) Disinfection of the surface and perimeter of the lesion. Needle is inserted through the normal tissue surrounding the lesion and punctured the middle layer of the lesion.(4) avoided injecting the drug into the blood vessel including suctioning before pushing the drug and then injecting the drug if there was no blood backflow. (5) Keeping the needle in the middle layer of the lesion and injected the drug slowly until the surface of the tumor was slightly white (**Figure 1**). (6) We determined the dose of the drug according to the size of the IHs and the patient’s weight. (7) The puncture point is pressurized to stop bleeding for 2 minutes, and Mucopolysaccharide Polysulfate Cream will be applied to the entire surface of the tumor. Parents are required to take care of the child to avoid friction and trauma to the lesion. Each course of treatment is divided into 5 consecutive days of injections, called low-dose injections, and the total dose per course of treatment did not exceed 2 mg/kg. Patients were reviewed 4 weeks after each course of treatment, and the doctor determined whether subsequent injection treatment was necessary after evaluating the symptoms and complications.

At the outpatient reexamination 4 weeks after each course of treatment, photographs of the lesions were taken, and ultrasound was used to determine whether the next injection treatment was needed. All images were evaluated individually by two specialized doctors. According to the criteria of Achauer et al., treatment effectivity was divided into four grades according to the percentage of regression: poor =<25%; moderate = 26% to 50%; good = disease 51% to 75%; and excellent = 76% to 100%.

Statistical analysis was conducted with the Statistical Package for the Social Sciences 24.0 (standard version IBM SPSS. USA). Baseline characteristics of patients and IHs are described in percent and mean. The Student t-test was used to compare mean/median values in the quantitative data analysis, while the x^2^ test was used for qualitative data. P-value<0.05 was considered significant.

## Results

The patients had a median age of 5.82( $\bar{\text{x}}$ +6.54)months. The sex ratio was 112:256(male: female). Mixed IHs (66.3%) was the most common, followed by deep IHs at 9.2% and superficial IHs at 24.5%. In total, 63(17.1%)lesions were on the head,22(6.0%)lesions were on the face, 5(1.4%)lesions were on the nose,18(4.9%)lesions were on the neck, 12 (3.3%) were on the lips, 98 (26.6%) were on the extremities, 150(40.7%)lesions were on the trunk. The mean diameter of the lesions was 2.67 cm, the thickness was 0.81cm. Information regarding baseline characteristics is shown in **Table 1**.

**Table 1:** Baseline characteristics of 368 infantile hemangiomas.

Characteristics		n (%)
Patients		368(100)
Sex		
	Male	112(30.4)
	Female	256(69.6)
Age at the time of first injection treatment (months)	Median ( $\bar{\text{x}}$ + x )	5.82 ( $\bar{\text{x}}$ +6.54)
Diameter of IH (cm)	Median ( $\bar{\text{x}}$ + x )	2.67 ( $\bar{\text{x}}$ +1.61 )
Deep of IH (cm)	Median ( $\bar{\text{x}}$ + x )	0.81 ( $\bar{\text{x}}$ +0.49 )
Lesion location		
	Head	63(17.1)
	Face	22(6.0)
	Nose	5(1.4)
	Neck	18(4.9)
	Lip	12(3.3)
	Extremity	98(26.6)
	Trunk	150(40.7)
Lesion type		
	Superficial	90(24.5)
	Mixed	244(66.3)
	Deep	34(9.2)
No. of injection(course)	Median (IOR)	1.68(1-4)

All known adverse reactions were recorded by us during the intralesional injection treatment and 1 month of follow-up, and no serious side effects were shown (**Table 2**). A total of 18 children had mild to moderate reactions, including 2 cases with ulcers, which appeared in superficial and mixed IHs during the first and second courses of treatment. They all appeared within 7 days after the end of one injection course and recovered after daily dressing changes and oral propranolol treatment. 3 cases of long-term swelling appeared in the superficial and mixed IHs during the third course of treatment. The lesions swelled immediately after injection treatment, and the swelling continued for more than 2 weeks after the treatment of Mucopolysaccharide Polysulfate Cream. Among the 3 children with skin allergy, 2 cases had multiple erythema on the chest and back, and 1 cases had multiple rashes all over the body. However, these erythema and rashes did not worsen and gradually returned to normal without treatment. Two patients experienced slight fever and temporary flushing occurred in 9 children, returned to normal without treatment.

**Table 2:** Characteristics of 18 infantile hemangiomas patients with complications.

	Ulceration	Prolonged swelling	Skin Allergy	Fever	Flushing
Lesion type	2	2	3	2	9
Superficial	1	0	2	1	5
Mixed	1	2	1	0	4
Deep	0	0	0	1	0
No. of injections					
1 course	1	0	1	0	5
2 course	1	0	1	2	2
3 course	0	2	1	0	2

All patients received low-dose lauromacrogol injections with and the median duration of injection treatment was 1.68 courses (range 1-4). The efficacy was evaluated 1 month after treatment and no other treatment was performed. 226(61.4%) achieved excellent regression. 108(29.4%) achieved good regression. 24(6.5%) achieved complete moderate regression. 10(2.7%)achieved poor regression (**Table 3**).

**Table 3:** Clinical response to sclerotherapy in infantile hemangiomas.

Achauer et al. as standard		n (%)
	Poor,<25% regression	10(2.7)
	Moderate,25–50% regression	24(6.5)
	Good,50–75% regression	108(29.4)
	Excellent, 75–100% regression	226(61.4)

**Figure 2:**
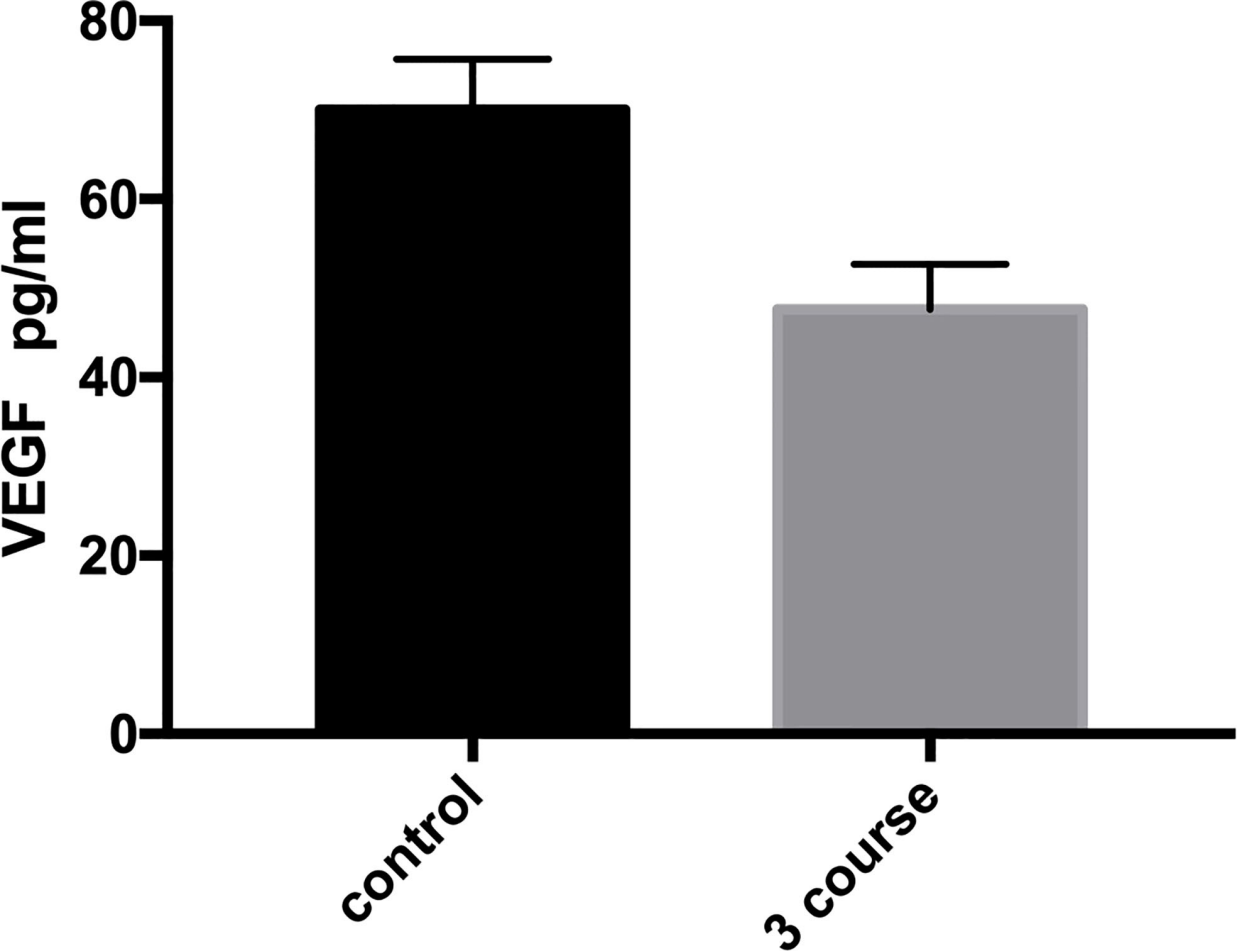
Relationship between VEGF and the course of injection in infantile hemangioma (IH). VEGF decreased significantly after 3 courses of treatment. P=0.0043.

A total of 68 cases with more than 3 courses of injection were tested for VEGF, and 40 children completed follow-up. Before the first treatment, the median VEGF level was 104.12pg/ml, however, after 3 courses of treatment, the level significantly decreased to 28.982pg/ml (P=0.0043). This indicates that the novel sclerosing agent injection treatment in this study can reduce VEGF level (**Figure 2**).

## Discussion

IHs has a unique evolutionary process starting with benign proliferation, followed by a slow regression phase can last 5 to 10 years (11). Although IHs tend to regress spontaneously, a range of complications can also develop, leading to pain, eating, and sleep problems that require early intervention (12). About half of the hemangioma will have residual tissue after spontaneous regression. The most common sequelae are fibrofatty residue and telangiectasia, requiring early intervention (13).

There are currently many treatment methods for hemangioma (2, 14), including propranolol, laser, freezing, glucocorticoid, vincristine, polidocanol (10) and pingyangmycin (15). Propranolol is considered first-line treatment for IHs, but it can cause complications including hypoglycemia, intractable diarrhea, hypotension, and some sequelae, including fibrofatty residue and telangiectasia (16). Due to these adverse effects, local injections directly into lesions may be beneficial.

Sclerotherapy is a simple, easy and effective method. It has been confirmed that intravascular sclerotherapy can effectively promote the regression of hemangioma and reduce residual lesions (17). In our study, 1 month after the last treatment, 97.3% of IHs responses were effective, and 61.4% of IHs had excellent regression results. Sclerotherapy destroys the cell membrane, leading to thrombosis, vascular lumen occlusion and irreversible damage to the vascular endothelium (10). In 1938, Kessler first summarized the advantages and disadvantages of this type of treatment. There are many types of sclerosing agents (18), polidocanol is the treatment of choice due to its anesthetic effect, low toxicity and low risk of allergic reactions. Lauromacrogol produced in China since 2008 promotes regression of IHs, but complications have been reported including ulcers, swelling, fever, infection, skin reactions, pain. In this study, we used a novel injection therapy of daily low-dose lauromacrogol to promote the regression of lesions and reduce complications by lowering the drug concentration. In the course of treatment, under the condition of not returning blood after aspiration, low concentrations of lauromacrogol are injected into the tissue rather than the intravascular site to avoid ectopic embolism, which may lead to tissue ischemia and necrosis if the drug enters normal blood vessels. Previous studies have reported that the most common complication of sclerotherapy is ulceration, but this study found that ulceration and fever were the two least common complications. Probably, a low concentration of the sclerosing agent may reduce its destructive effect. At the same time, drug injection into the tissue space appears to reduce the possibility of arterial embolism and phlebitis.

During the evolution of IHs, VEGF is a powerful angiogenic factor that plays an essential role in vascular development and abnormal angiogenesis, promoting proliferation, adhesion, migration and differentiation (19). Both *in vivo* and *in vitro* experiments have demonstrated that serum VEGF significantly increases in the proliferative phase (6, 20). The VEGF signaling pathway is involved in the pathogenesis of IHs and regulates angiogenesis. Propranolol can cause vasoconstriction, tumor growth inhibition, angiogenesis inhibition, and induce apoptosis (21). Makkeyah et al. found that VEGF levels decreased 3 months after propranolol treatment of IHs, leading to the inference of an intermediate effect of propranolol through down-regulation of VEGF, leading to proangiogenic cascades and inhibition of angiogenesis (6). Some studies have demonstrated, through long-term observation that by injecting sclerosant, the invasive growth can be inhibited, and the natural regression process can be accelerated (10). In our study, VEGF levels decreased three course after lauromacrogol treatment of IHs. Therefore, we speculate that low-dose injection therapy may block the growth of IHs by down-regulating VEGF and promote their transition into remission.

In conclusion, the continuous, osmotic and low-dose injection of lauromacrogol for the treatment of IHs appears to be safe and effective. Further studies are needed to confirm our findings.

## Data availability statement

The original contributions presented in the study are included in the article/supplementary material. Further inquiries can be directed to the corresponding author.

## Ethics statement

The studies involving human participants were reviewed and approved by ethics committee of Children’s Hospital Affiliated to Shandong University. Written informed consent to participate in this study was provided by the participants’ legal guardian/next of kin.

## Author contributions

WC, study design, performed measurements, statistical analysis, and manuscript preparation. SD, statistical analysis and manuscript preparation. JS, study design and manuscript preparation. WL, XZ and ZL, manuscript preparation. LG, corresponding author, study design, and manuscript preparation. All authors contributed to the article and approved the submitted version.

## Funding

The research is supported by the Fund No.: Science and Technology Plan Project 2021-1-38 of Jinan Health Commission and Clinical Medicine Science and Technology Innovation Plan 202019063.

## Conflict of interest

The authors declare that the research was conducted in the absence of any commercial or financial relationships that could be construed as a potential conflict of interest.

## Publisher’s note

All claims expressed in this article are solely those of the authors and do not necessarily represent those of their affiliated organizations, or those of the publisher, the editors and the reviewers. Any product that may be evaluated in this article, or claim that may be made by its manufacturer, is not guaranteed or endorsed by the publisher.
